# Pharmacological and Biological Antiviral Therapeutics for Cardiac Coxsackievirus Infections

**DOI:** 10.3390/molecules16108475

**Published:** 2011-10-11

**Authors:** Henry Fechner, Sandra Pinkert, Anja Geisler, Wolfgang Poller, Jens Kurreck

**Affiliations:** 1 Department of Applied Biochemistry, Institute of Biotechnology, Technische Universität Berlin, Gustav-Meyer-Allee 25, 13355 Berlin, Germany; Email: sandra.pinkert@charite.de (S.P.); jens.kurreck@tu-berlin.de (J.K.); 2 Department of Cardiology & Pneumology, Charité – Universitätsmedizin Berlin, Campus Benjamin Franklin, Hindenburgdamm 30, 12200 Berlin, Germany; Email: anja.geisler@charite.de (A.G.); wolfgang.poller@charite (W.P.)

**Keywords:** coxsackievirus, myocarditis, soluble receptors, RNA interference, antiviral drugs

## Abstract

Subtype B coxsackieviruses (CVB) represent the most commonly identified infectious agents associated with acute and chronic myocarditis, with CVB3 being the most common variant. Damage to the heart is induced both directly by virally mediated cell destruction and indirectly due to the immune and autoimmune processes reacting to virus infection. This review addresses antiviral therapeutics for cardiac coxsackievirus infections discovered over the last 25 years. One group represents pharmacologically active low molecular weight substances that inhibit virus uptake by binding to the virus capsid (e.g., pleconaril) or inactivate viral proteins (e.g., NO-metoprolol and ribavirin) or inhibit cellular proteins which are essential for viral replication (e.g., ubiquitination inhibitors). A second important group of substances are interferons. They have antiviral but also immunomodulating activities. The third and most recently discovered group includes biological and cellular therapeutics. Soluble receptor analogues (e.g., sCAR-Fc) bind to the virus capsid and block virus uptake. Small interfering RNAs, short hairpin RNAs and antisense oligonucleotides bind to and led to degradation of the viral RNA genome or cellular RNAs, thereby preventing their translation and viral replication. Most recently mesenchymal stem cell transplantation has been shown to possess antiviral activity in CVB3 infections. Taken together, a number of antiviral therapeutics has been developed for the treatment of myocardial CVB infection in recent years. In addition to low molecular weight inhibitors, biological therapeutics have become promising anti-viral agents.

## 1. Introduction

Myocarditis is defined as a subclinical inflammation of the heart muscle and may be induced by infectious, toxic or immunologic agents. Among the different infectious pathogens viruses are the most common causes of myocarditis [1] and serological studies, nucleic-acid hybridization and PCR-based studies of endomyocardial biopsy and autopsy specimens have shown that enteroviruses represent one of the most common groups of viruses detected in the myocardium [2]. Although myocarditis is a frequent disease, it often goes unrecognized. Based on more than 12,500 randomly selected routine autopsies performed over a 10 year period about 1% of the population were found to have lymphocytic myocarditis [3]. In most cases myocarditis is detected in the group of young adults between the ages of 20 and 39 [4].

Coxsackieviruses belong to the family *Picornaviridae* and the genus enterovirus. The viruses have a positive-stranded RNA genome of about 7.4 kb encoding a monocistronic polyprotein [5] which is processed into mature peptides during translation by viral proteases [6]. Four capsid proteins (VP1 to VP4) and seven nonstructural proteins (2A, 2B, 2C, 3A, 3B, 3C, and 3D) are found during coxsackievirus infection of cells [7]. Based on different organ tropism and differences in organ damage observed in mice, coxsackieviruses can be classified into two groups. The group A coxsackieviruses (CVA) has 23 members, whereas the group B coxsackieviruses (CVB) has only six members [8,9]. Coxsackieviruses commonly induce mild disease, but under some circumstances, which seem to depend on genetic and individual predispositions, the viruses overcome local host defenses and can induce severe infections of the heart, pancreas, and brain [9]. The CVB group includes serotypes, in particular CVB3, that are frequently associated with infections of the heart. The course of CVB3 heart infections in patients may be different. Typically CVB3 induces acute myocardial infections and, in most cases, patients undergo a complete recovery. Fulminant infections, however, can occur and can result in sudden death. If the virus cannot be eliminated by host immune defense mechanisms, a persistant CVB3 infection and dilated cardiomyopathy (DCM), one of the leading causes for heart transplantation, can be developed. The incidence of enteroviral infections in the hearts of patients with myocarditis and DCM is variable but infection rates of up to 40% have been observed [2,10,11]. Although DCM has multiple causes, there is evidence that 30% of DCM patients are infected with enteroviruses [12,13]. 

The pathogenic mechanisms of CVB3 induced myocarditis are yet not fully understood. That enteroviruses play an active role in development of myocarditis in humans, especially in fulminant and acute courses of infections, is supported by detection of enteroviral replicative intermediates (minus-strand enteroviral RNA) in 50% of patients with left ventricular dysfunction and suspected clinical myocarditis [10]. In contrast, in patients with idiopathic DCM and chronic coronary disease (CCD), representing end-stage cardiac diseases, 36% and 33% of the patients were positive for enterovirus genomes, but only a small percentage (6.5%) of them had an active enteroviral replication in the myocardium. Moreover, no fully replicative virus could be isolated from myocardium of the patients [14]. In chronic heart infections, coxsackieviruses persist in a latent form and animal models have shown that virus persistence can result from production of stable double-stranded RNA [15] or is associated with deletions in the 5’-non-translated region of the viral genome [16]. Based on these observations, it was important to determine whether replication deficient coxsackievirus variants are able to induce DCM. To prove this Wessely *et al*. generated transgenic mice that expressed a replication restricted CVB3 cDNA exclusively in the heart. These animals expressed the coxsackieviral genomes in their cardiomyocytes at low-level without forming infectious virus particles. Histopathological analysis of transgenic hearts revealed typical morphologic features of myocardial interstitial fibrosis and degeneration of myocytes, resembling DCM in humans. Functionally these changes were accompanied by decreased systolic functions and excitation-contraction coupling abnormalities similar to pressure overload models of DCM [17]. Thus, this study confirmed that non-replicative coxsackieviruses are sufficient to induce DCM. 

Studies in *in vitro* and *in vivo* models carried out over the last 25 years have contributed significantly to elucidating important aspects of the molecular pathogenic mechanisms of the cardiac coxsackievirus infection. Based on these studies, it is clear that both direct virus induced injury as well as immune and autoimmune mechanisms triggered by viral infection are involved in development of coxsackievirus induced myocarditis [18,19]. The impact of direct virus induced cytotoxicity is supported by the fact that CVB3 RNA is detected at all stages of CVB3 heart infections [20] and that the virus replication correlates with negative clinical outcome, suggesting that continued replication of the virus is involved in the progression of the disease [21]. Furthermore, it has been shown that enterovirus induced cytotoxicity is directly related to expression of viral proteins. CVB3 expresses two viral proteases, 2A and 3C. Both can induce apoptosis through activation of the extrinsic caspase-8-mediated pathway and the intrinsic mitochondria-mediated apoptosis pathway [22]. The protease 2A also cleaves the eukaryotic translation initiation factors (eIFs) eIF4GI and eIF4GII, leading to cessation of host protein synthesis [23,24] and is able to cleave dystrophin in CVB3 infected cultured myocytes and in infected mouse hearts [25,26]. The latter is thought to play a role in release of the virus from the myocyte [27] since viral infection is increased in the absence of dystrophin [28]. More importantly, impaired dystrophin functions lead to the disruption of the extra-sarcomeric cytoskeleton and loss of transmission of mechanical force to the extracellular matrix [29,30] which seem to be important factors contributing to the development of DCM [31,32,33,34]. 

Although therapeutic approaches aiming at modulation or inhibition of the immune system, such as immunoglobulin therapy, immunoabsoption or specific antibody therapy hold also promise for the treatment of myocardial enteroviral infections [35,36,37], this review will focus on direct pharmacological and biological therapies targeting specific points of the virus replication cycle to inhibit coxsackievirus-induced myocarditis.

## 2. Pharmacologically Active Low Molecular Weight Substances

Infection and replication of any virus, including CVB3, starts with interaction of the virus and its cellular receptor on the host cell, and ends with the release of viral progeny. Every step during this life cycle is a potential target for inhibiting viral infection. Based on their targets, pharmacologically active low molecular weight substances that are able to selectively inhibit the replication of coxsackieviruses can be divided into three groups. The first group consists of small molecules witch interfere with the viral capsid and prevent attachment/internalization or uncoating of the virion in the host cell. Substances of the second group inhibit viral replication by directly interfering with viral proteins and those of the third group interact with cellular proteins which play an important role in viral replication.

### 2.1. Inhibition of Viral Attachment, Internalization or Uncoating

The first essential step of viral infection is the interaction of the viral capsid with the receptor on the surface of the host cell. Coxsackievirus capsids are assembled from 60 identical protomers, each composed of the four structural proteins VP1–VP4. The viral shell is formed by VP1 to VP3, and VP4 lies on the inner surface and establishes a connection between the capsid and the RNA genome [7]. The surface of the virion shows a five-fold axis of symmetry, surrounded by a large depression termed the “canyon” which contains a hydrophobic pocket [38]. The coxsackievirus and adenovirus receptor (CAR) is the primary receptor of CVB [39,40] and its binding to the pocket on the canyon floor of CVB mediates the internalization of the virus into the host cell [41].

**Figure 1:**
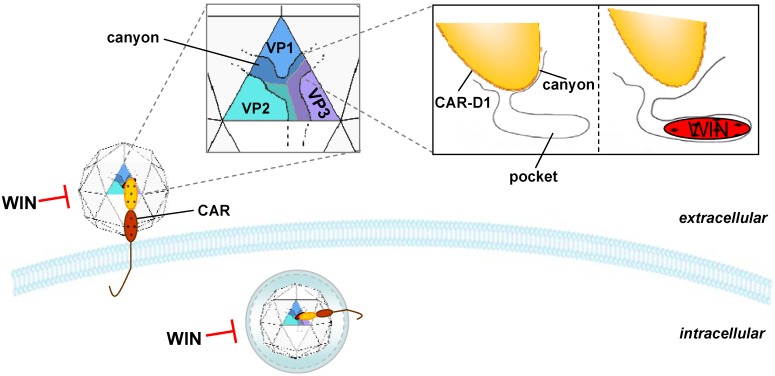
Inhibition of coxsackieviruses by WIN compounds. WIN compounds bind to the canyon pocket of CVB3 thereby inhibiting the binding of the virus to the cellular receptor CAR. WIN compounds also inhibit the uncoating and release of the viral RNA into the cytoplasm.

Several drugs have been developed that selectively inhibit the interaction of CVB with CAR. The WIN compounds are antiviral drugs interacting with the hydrophobic pocket at the bottom of the canyon [42] (Figure 1). They were discovered initially by the Sterling-Winthrop Research Institute and first used for inhibition of rhinovirus infections [43]. These small molecules inhibit interaction of the virion with the cellular receptor molecule and thus prevent virus binding to the target cell receptors [44]. Furthermore, binding of specific WIN molecules in the pocket results in an increase in protein rigidity and stabilizes the entire viral capsid against enzymatic degradation so that uncoating and release of viral RNA into the cytoplasm is prevented [45,46,47]. In the last 25 years many WIN based drugs have been tested regarding their inhibitory effects against different members of the picornavirus family such as rhinovirus [48], poliovirus [49], echovirus [50], and enterovirus [51]. WIN 54954, a broad-spectrum anti-picornaviral drug, was one of the first WIN compounds to be clinically tested. Its effectiveness against human rhinovirus, echovirus 9 and also enterovirus infections has been shown *in vitro* and *in vivo* [51,52,53,54]. *In vitro* studies demonstrated that WIN 54954 can reduce picornavirus titers by 1 to 2 orders of magnitude [51,52]. *In vivo* administration in mice experimentally infected with CVB3 led to a reduction of the proportion of cardiomyocytes containing viral RNA by about 90% and significantly inhibited cardiomyocyte apoptosis [55]. Moreover, administration of the drug resulted in complete protection against mortality of CVB3 in infected mice [56]. The latter study revealed that a strong decrease of virus induced mortality occurred when the treatment was started concomitant with CVB3 infection. When onset of therapy was delayed for one day, 85% survival was observed, whereas in the non-treatment group no animal survived, demonstrating significant, if no longer complete, protection from the virus. A therapeutic effect was still notable when treatment was initiated 4 days after virus inoculation [57]. Thus WIN 54954 therapy was almost fully effective within 24 hours after CVB3 infection, but the beneficial effect declined over time [57]. WIN 54954 treatment did not, however, abrogate the inflammatory reaction in the myocardium in CVB3 infected animals [55,56]. As shown in a phase 1 clinical trial against rhinovirus infections and in a myocarditis model of mice infected with coxsackievirus A9, low doses of WIN 54954 were well tolerated [53,58]. At higher doses of 100 to 200 mg/kg/day, however, neurological toxicity was observed in the murine myocarditis model *in vivo* [53]. Moreover, the compound is rapidly metabolized and induces reversible hepatitis, which made this compound uninteresting for further studies in the end [59].

WIN 63843 (pleconaril) represents a novel, orally available, systemically-acting compound of the WIN series with a high oral bio-availability and long plasma half-life. The compound has a broad spectrum of antiviral activity against clinical isolates and prototypic strains of enteroviruses, including CVB1–CVB5, *in vitro* [60,61]. Pleconaril has been more extensively evaluated in clinical studies than any other anti-picornavirus drug and shows clear efficacy in the treatment of various picornavirus-associated illnesses, whereas side effects are rare [4,62,63,64]. In one study Rotbart *et al*. investigated the therapeutic response after pleconaril treatment in a follow up study in 38 patients with potentially life-threatening enterovirus infections. About 80% of the patients had a clinical response temporally associated with pleconaril therapy and to a similar degree patients showed virological, laboratory and radiological responses [4]. Clinical trials, however, revealed that therapeutic efficacy of pleconaril depends on the virus species targeted. A significant therapeutic effect was apparent during treatment of rhinovirus and several enterovirus infections [59,65,66]. In 2002 the Federal Drug Administration (FDA) of the USA declined to approve pleconaril for treatment of the common cold, as the panel remained unconvinced about the drug’s safety profile [67]. Pleconaril has not yet been used in clinical trials explicitly for the treatment of enterovirus induced myocarditis. *In vivo* studies carried out by Pevear *et al*., however, demonstrated an efficient antiviral efficacy of the compound in enterovirus induced mouse myocarditis. Mice showed reduced mortality and a reduction of the CVB3 titer in the heart by about 4 to 7 orders of magnitude [60]. 

Several studies demonstrated naturally occurring mutants resistant to pleconaril and, moreover, pleconaril treatment seems to result in rapid emergence of resistant CVB3 mutants [68,69]. Such mutants predominantly contain a single amino acid substitution, Ile-1092 ➔ Leu/Met in the hydrophobic pocket of the canyon which prevents efficient binding of the compound, while it does not impair binding of the virus to cellular receptors [69]. In order to overcome virus resistance, new pleconaril derivatives have recently been synthesized and successfully tested against pleconaril-resistant mutants [70].

In the last few years several additional new drugs have been developed which are functionally similar to the WIN compounds and have good antiviral efficacy against rhinovirus infection but less activity against coxsackievirus infections [71]. Thus, the search for more potent, highly bio-available compounds with a broad antiviral spectrum and high efficacy goes on.

### 2.2. Inhibition of Viral Replication

**Figure 2:**
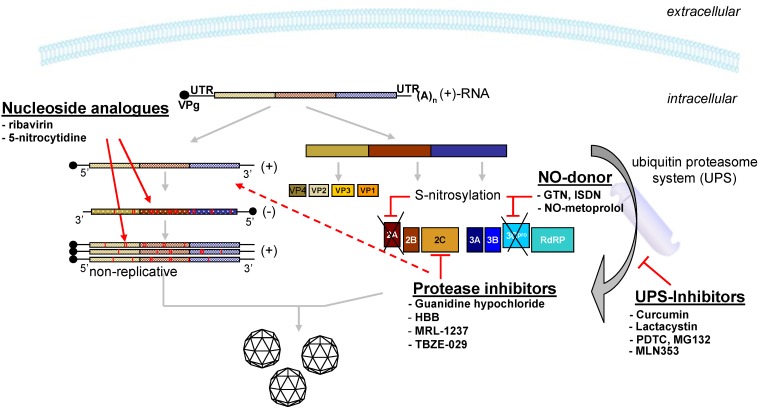
Pharmacologically active low molecular weight substances and their targets in the coxsackievirus replication cycle.

The second class of picornavirus inhibitors comprises drugs that interfere with viral proteins during replication. These compounds interact with non-structural proteins, viral proteases or the RNA-dependent RNA polymerase and thereby inhibit viral replication in infected cells. A series of compounds, such guanidine hypochloride, HBB, MRL-1237 and TBZE-029 interact with the viral protein 2C, resulting in inhibition of the viral RNA synthesis and leading to protection of cells from virus-induced cell lysis [71,72] (Figure 2). Their antiviral activity against coxsackieviruses was only investigated in *in vitro* experiments. These experiments, however, revealed that a single mutation in the viral 2C protein is sufficient to confer resistance against the antiviral treatment [71]. 

Nitric oxide (NO) donors form another class of molecules which interfere with the functions of viral non-structural proteins (Figure 2). They inhibit enterovirus proteases 2A and 3C by S-nitrosylation [73,74]. Both proteases are involved in host cell shut-off and lead to the development of the cytopathic effects [75,76,77]. The NO donors GTN and ISDN are able to inhibit coxsackievirus proteases and induce a significant antiviral effect *in vitro.* Furthermore, GTN significantly reduced signs of myocarditis after administration by decreasing immune cell infiltration and virus-induced fibrosis up to day 14 post-infection (p.i.) [78]. In another study the NO-donor was NO-metoprolol, a novel NO-releasing derivative of the β1-selective adrenergic receptor agonist metoprolol. It has been shown that metoprolol improves the survival, remodeling, fibrosis and left ventricular systolic function in a knock-in mouse model for inherited dilated cardiomyopathy [79]. In the CVB3 myocarditis mouse model treatment with NO-metoprolol displays an enhanced therapeutic benefit compared to metoprolol, with significant reduction of viral RNA copy numbers, body weight loss, infiltration and fibrosis score [80].

Several nucleoside analogues were analyzed with respect to their anti-picornaviral potency. The most intensively studied drug is ribavirin, a synthetic nucleoside, structurally related to inosine and guanosine. When ribavirin is incorporated into viral RNA it induces mutations that can be lethal for the virus (Figure 2). It has been shown that ribavirin has a broad antiviral activity against several RNA viruses *in vivo*, e.g., RSV, measles, lassa, and hepatitis C viruses [81,82,83]. *In vitro* activity of ribavirin against enteroviruses has been demonstrated by several groups [84,85,86], and treatment of murine CVB3 myocarditis led to significantly decreased myocardial virus titer, inflammation, necrosis and myocardial calcification [86]. One side effect of ribavirin treatment, however, is hemolytic anemia [87], so ribavirin may be contraindicated in patients with acute and chronic myocarditis. In a further study, Harki *et al*. synthesized cytidine analogues and evaluated them for antiviral activity. One of the tested molecules, a 5-nitrocytidine, decreased viral titer in CVB3 infected cells with 12-fold higher efficiency than ribavirin, but so far the results of *in vivo* studies, if any, have not been reported [88].

### 2.3. Inhibition by Interfering with Cellular Proteins

A third group of antiviral drugs includes molecules which interact with cellular proteins and thereby interfere with viral replication. Several studies have shown that ubiquitination is required for effective replication of different viruses, among them coxsackievirus [89,90,91]. The ubiquitin-proteasome system (UPS) is a major intracellular pathway for protein degradation, with over 80% of cellular proteins being recycled through this pathway. UPS thus plays a key role in the regulation of a variety of fundamental cellular processes, such as signal transduction, cell cycle regulation, apoptosis, antigen processing, transcriptional regulation and DNA repair [92,93,94]. In the last few years several inhibitory substances targeting the UPS were analyzed for their ability to inhibit coxsackievirus infections (Figure 2). Pyrrolidine dithiocarbamate (PDTC) and curcumin, a natural polyphenolic compound extracted from *Curcuma longa*, reduced coxsackieviral RNA synthesis, protein expression and progeny release up to 170-fold and 20-fold, respectively, *in vitro* [95,96]. Other UPS-inhibitors, the peptide-aldehyde MG132 and lactacystin, reduced CVB3 infectivity in the murine cardiomyocyte cell line HL-1 and increased cell viability [90]. Treatment of CVB3 infected mice with the proteasome inhibitor MLN353, however, resulted in an only slight decrease of viral-induced mortality and myocardial injury. Significant inhibition of viral replication in the heart was not detectable [97]. Although UPS inhibitors may be potential therapeutic tools for treatment coxsackievirus infection their relatively low efficiency and possible drug-induced side effects may limit their use for treatment of coxsackievirus infections in humans. 

In summary, over the last 25 years many pharmacologically active low molecular weight substances have been developed, concomitant with the improvement of our knowledge of picornavirus structure and replication cycle. Adverse properties in bio-availability and safety of these drugs, rapid development of escape mutants and, in part, low efficacy currently limits their use in treatment of myocardial coxsackieviral infections in humans.

## 3. Interferons

Interferons (IFNs) belong to the group of the cytokines and have antiviral and immunomodulating activities. Suspected from early investigations demonstrating the release of cytokines from CVB3 infected human monocytes [98], the role of IFNs in CVB3 infections became obvious when IFN-β knockout mice proved to be highly susceptible to CVB3 infections and developed a breakdown and disruption of cardiomyocytes [99]. 

*In vivo* and *in vitro* studies demonstrated the antiviral efficacy of IFN treatment for coxsackievirus infections of the heart. Heim *et al*. showed antiviral activity of recombinant IFN-β and IFN-γ and ribavirin and natural human IFN-α co-treatment in a CVB3 carrier state infection of cultured human myocardial fibroblasts *in vitro* [85,100]. In another study, Wang *et al*. confirmed antiviral and cardio-protective efficacy of murine IFN-β and IFN-α_2_ in an CVB3 induced mice myocarditis model *in vivo* [101]. The potency of IFNs for treatment of myocardial enteroviral infections in humans was demonstrated several years ago. Twenty-two patients with entero- or adenoviral myocardial persistence were treated with IFN-β over a period of six months. Enteroviral genomes were eliminated from all patients. The viral clearance was paralleled by a significant decrease of left ventricular end diastolic and end systolic diameters of the heart and by an improvement of left ventricular function. Moreover, IFN-β was well tolerated in this study. Patients only showed flu-like side effects during the first 3 weeks of IFN-β treatment that could be efficiently suppressed by non-steroidal anti-inflammatory drugs. Further investigations of the therapeutic potential of IFN-β are currently being carried out in an ongoing multicenter randomized Betaferon^®^ (Bayer Schering Pharma AG) study in patients with chronic viral cardiomyopathy (BICC study) [102].

## 4. Antisense Oligonucleotides

Antisense approaches have proven to be promising strategies to inhibit viruses. Conventional antisense oligonucleotides are single-stranded molecules composed of DNA monomers and modified variants thereof. These antisense oligonucleotides bind to a complementary (virus) RNA and prevent its translation or induce its degradation by RNase H [103]. The only antisense oligonucleotide that has been approved by the regulatory authorities to date is fomivirsen, a phosphorothioate-modified DNA oligonucleotide which was used to treat retinitis induced by cytomegalovirus, but was discontinued since the drug’s market shrank [104]. 

Antisense oligonucleotides have also been considered as novel tools to inhibit CVB3. In an initial screening, seven antisense oligonucleotides were designed to target the 5’ and 3’ UTR of CVB3 and evaluated with respect to their antiviral activity in HeLa cells [105]. Phosphorothioate-modified oligodeoxynucleotides were used that are resistant to degradation by cellular nucleases. Efficient virus inhibition was achieved with antisense oligonucleotides directed against either of the two untranslated regions. The most efficient antisense molecule targeting the 3’ UTR was subsequently used to inhibit CVB3 in HL-1 cells and *in vivo* [106]. This antisense oligonucleotide strongly inhibited viral RNA and protein synthesis in cell culture and its intravenous administration into A/J mice decreased the viral titer by 0.5 log_10_. To improve the *in vivo* delivery, a cell-penetrating arginine-rich peptide was conjugated to a morpholino oligomer [107]. Since morpholino oligomers do not induce RNase H cleavage of the target RNA, their mechanism of action is based on a steric blockade of the target RNA function. Intravenous administration of the oligomer once prior to and once after CVB3 infection resulted in an enhanced, approximately 2 order of magnitude decrease in viral titer and in significantly less damage to cardiac tissue.

## 5. RNA Interference

**Figure 3:**
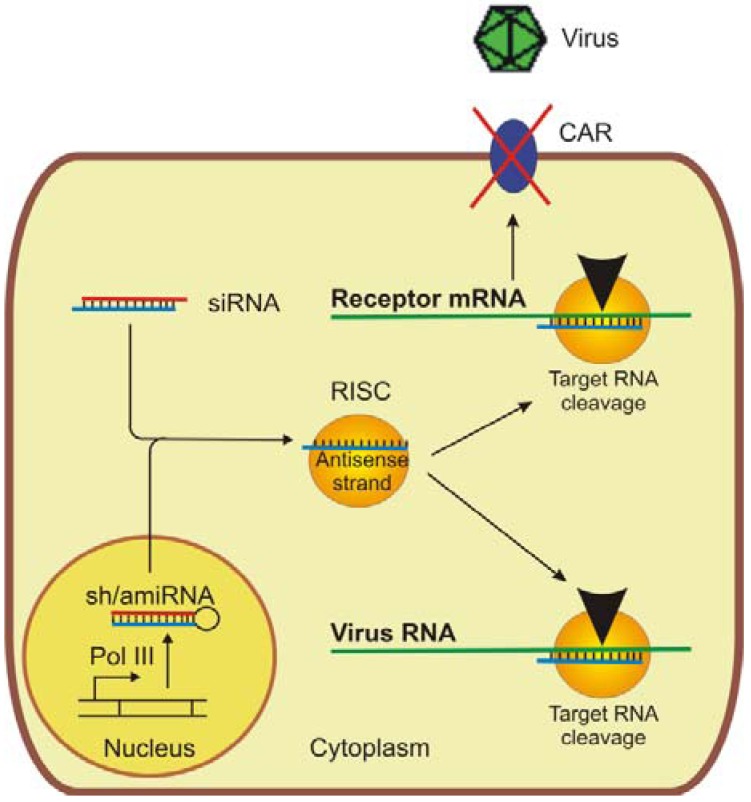
Antiviral applications of RNAi. RNAi can either be induced by chemically synthesized small interfering RNAs (siRNAs) or by vector expressed short hairpin RNAs (shRNAs) or artificial microRNAs (amiRNAs), respectively. The activated RNA-induced silencing complex (RISC) contains only the antisense strand of the double-stranded RNA. The RNAi approach can either be directed against the virus itself or against the virus’s receptor, whose silencing will prevent virus entry into the cell.

A decade ago short double-stranded RNA molecules were found to efficiently inhibit gene expression in mammalian cells [108]. These so-called small or short interfering RNAs (siRNAs) are usually 21 nucleotides in length, 19 of which form a duplex. After introduction into cells, siRNAs elicit efficient induction of post transcriptional gene silencing known as RNA interference (RNAi). They are incorporated into a multimeric protein complex named RNA-induced silencing complex (RISC). While one of the two RNA strands is discarded, the antisense strand guides RISC to the complementary target RNA and induces its endonucleolytic cleavage (Figure 3). Comparative studies revealed a significantly higher efficacy of RNAi compared to the application of antisense oligonucleotides [109].

The duration of gene silencing mediated by siRNAs is limited to a few days at most. Therefore, an alternative approach was developed to express self-complementary short hairpin RNAs (shRNAs) intracellularly. These shRNAs are exported from the nucleus and processed into siRNAs, which can then be incorporated into RISC as described above. In addition, the shRNA strategy opens up the possibility of delivering expression cassettes into the target cells by viral vectors. The most commonly used vectors are based on retroviruses (including lentiviruses), adenoviruses and adeno-associated viruses (AAV) [110].

Soon after its discovery, RNAi was considered a novel option to treat viral infections. Successful RNAi applications have been reported for most classes of medically relevant viruses including HIV-1, HBV, HCV, SARS-coronavirus, influenza virus und poliovirus [111,112]. Some of these approaches have already reached the stage of clinical testing. While RNAi-mediated therapies against HIV-1 and HBV are based on shRNA expression systems [113], the most advanced clinical phase II trial makes use of chemically synthesized siRNAs against RSV, which are administered by a nasal spray [114].

### 5.1. RNA Interference against CVB3

Because of the highly efficient nature of RNAi, it comes as no surprise that RNAi has been evaluated as novel strategy to inhibit CVB3 [115,116,117,118,119,120]. All of the initial studies showed that only siRNAs against the coding region of the virus genome revealed significant antiviral activity, but not those against the untranslated regions. In order to investigate the mechanism of RNAi-mediated inhibition of CVB3 in more detail, siRNAs were intentionally designed to target either the viral plus strand or the minus strand or both [121]. This study provided clear evidence that silencing of the viral plus-strand is the key to inhibit CVB3, most likely because the minus-strand might be inaccessible to the silencing machinery.

### 5.2. RNA Interference-Strategies to Prevent Viral Escape

A major problem for the long term inhibition of viruses is the emergence of escape mutants. This limitation, which is well known for conventional antiviral therapy with low-molecular weight drugs, is applicable to RNAi approaches as well. Virus escape as a consequence of the accumulation of point mutations in or close to the siRNA target site has been observed for various types of viruses, including poliovirus [122] and HIV [123,124]. Three counter-strategies have been followed to counter the problem of viral escape: (1) targeting of conserved regions of the virus genome; (2) combination of efficient antiviral siRNAs; and (3) silencing of host factors that are essential for the viral life cycle.

Mutations in highly conserved regions of the virus genome are likely to cause a loss of virulence. Comparison of silencing by siRNAs against different genomic regions of CVB3 revealed that targeting of nonstructural protein coding regions is superior to selecting structural protein coding regions, since enzymes often lose activity when mutations occur [125]. Several groups have initially directed siRNA against the 5’ untranslated region (5’UTR) of the CVB3 genome, which harbors the internal ribosome entry site (IRES). Interestingly, none of the tested siRNAs exerted significant antiviral activity [118,119,125,126]. A possible explanation for this unexpected finding is that tight structures such as the IRES are detrimental to siRNA-mediated gene silencing [127]. Only after laborious screening for accessible sites for complementary oligonucleotides in the 5’ UTR could siRNAs targeting the IRES be developed that were capable of inhibiting CVB3 [128]. The antiviral activity of the siRNA was improved further by its partial modification with locked nucleic acids (LNA), which have a high affinity towards complementary RNAs. Another highly conserved region of the CVB3 genome is the *cis*-acting replication element (CRE) located in the 2C protein coding region. An siRNA directed against this region conferred sustained protection against CVB3 and prevented the emergence of viable escape mutants [129]. Since the CRE sequence is identical in other enteroviruses such as echoviruses 6 and 7 and A-type as well as other B-type coxsackieviruses, the siRNA has a universal and persistent anti-enteroviral activity.

A widely employed strategy to minimize viral escape in conventional virus therapy is to combine various agents with antiviral activity. For HIV, this approach is known as highly active anti-retroviral therapy (HAART) or combined anti-retroviral therapy. The adaptation of this idea to RNAi is the combination of two or more active siRNAs or shRNAs. Combination of two shRNA expression cassettes in one vector was found to maintain silencing activity against mutated target RNAs of CVB3 in a reporter system, since the second shRNA can compensate for the loss of silencing activity of the shRNA directed against the mutated target site [115]. A systematic investigation of viral escape in cell culture revealed that cocktails of three siRNAs targeting distinct sites of the virus genome could maintain therapeutic efficacy, while virus inhibition with dual- or single-molecule-based RNAi was abrogated by viral escape [125].

In the long run, however, resistant mutants are likely to develop even against combinations of three or more site-specific siRNAs. Therefore, recently the idea was advanced to use a pool of siRNAs covering 3.5 kb of CVB3 genomic sequence [130]. The pool was generated by synthesizing a long double-stranded RNA covering the region encoding most of the non-structural proteins of CVB3, which was subsequently cleaved into siRNAs by recombinant Dicer. The pool was found to be significantly more effective than single-site siRNAs. Although this strategy can be expected to prevent viral escape, its therapeutic application is questionable since the antiviral agent consists of a heterogeneous mixture of hundreds of different siRNA molecules. It is currently unclear whether the pool of siRNAs will induce more severe off-target effects than single siRNAs, since it consists of numerous sequences, each of which can potentially regulate non-target RNAs. 

The third strategy for sustained inhibition of viral spread is silencing of genes of the host cells that are required by the virus to enter cells and replicate (Figure 3). The advantage of this approach is that viruses have a limited capacity to adapt to host cell changes. CVB3 initially attaches to the decay-accelerating factor (DAF), which serves as a co-receptor, prior to the virus entering the host cells via CAR. Whereas CAR is essential for cardiac CVB3 infection [131] most or all CVB2, 4, 6 as well as some strains of CVB1, 3 and 5 do not bind DAF [132]. As expected, silencing of CAR was found to prevent infection of the treated cells by CVB3 [119,133]. Whether CAR can be considered a therapeutic target is debatable, since an essential prerequisite for the medical application of this strategy would be that the factor is dispensable. CAR is located in the tight junctions of epithelial cells [132,134] and its constitutive knockout was found to result in an embryonic lethal condition associated with cardiac defects [135]. In contrast, animals with a conditional knockout of CAR at a later time point of embryonic development (E11) survived to adulthood and did not have evident cardiac abnormalities [136]. Very detailed investigations of heart function, however, revealed a block of atrio-ventricular conduction developed in the absence of CAR in the adult mouse heart, which may lead to arrhythmia [137,138]. This finding was confirmed more recently with conditional knockout mice that exhibited a complete atrio-ventricular block and various phenotypes in other organs as well [139]. It should, however, be noted that no direct conclusions can be drawn from knockout experiments for RNAi applications, since knock-out animals completely lack CAR while RNAi only results in a partial knockdown of target gene expression, possibly leaving enough to prevent these defects from occurring. 

Since CAR might have essential cellular functions, other host factors should be considered as targets for inhibitors that block CVB3 indirectly. In a recent study, an RNAi screen covering the druggable genome was carried out to identify cellular factors required for CVB3 infection of human cells [140]. With this approach, a set of genes could be identified whose depletion inhibited infection. Further studies will now be necessary to evaluate whether any of these genes are suitable candidates for the development of inhibitors with antiviral activity.

### 5.3. Chemical Modified siRNAs

Cellular delivery, potency and specificity of siRNAs can be improved by chemical modification of the double-stranded RNA [141]. In addition to LNA-modified siRNAs, which were mentioned above, siRNAs containing LNA, as well as unlocked nucleic acids (UNA), have been tested against CVB3 [142]. In this study, a design pattern could be elucidated in which the UNA monomers do not compromise the high antiviral siRNA activity. Further studies have shown that the introduction of UNAs into an siRNA minimizes off-target activity [143], which constitutes one of the major challenges associated with the clinical use of RNAi. A second major hurdle for the application of RNAi is efficient cellular delivery of the siRNAs. Attachment of lipophilic molecules such as cholesterol or receptor ligands like folate have been shown to improve, in some cases cell-type specifically, cellular uptake of a given siRNA, even in the absence of delivery agents. A folate-linked bacterial phage packaging RNA was used as a vehicle to deliver double-stranded RNA into HeLa cells, a folate receptor positive cancer cell line widely used as an *in vitro* model for CVB3 infection [144]. As a further development of this strategy, an artificial micro RNA (amiRNA) targeting the 3’ untranslated region of the CVB3 genome instead of standard siRNAs was used as the silencing mediator [145]. The amiRNA was designed to contain mismatches to the central region of the target site and was shown to tolerate mutations, thus having the potential to suppress viral escape mutants.

### 5.4. Treatment of CVB3 Infections in Animal Models

A major challenge in the development of new therapeutic strategies is the translation of knowledge gained with cell culture experiments to the *in vivo* situation. An siRNA targeting the 2A protease encoding genomic region was found to lead to significant reduction of viral tissue titers, attenuate tissue damage, and prolong survival in highly susceptible type I interferon receptor-knockout mice [116]. As outlined above, prolonged silencing can be achieved by continuous intracellular generation of shRNAs from expression cassettes. As a proof-of-principle for this approach, plasmid-derived shRNAs were used to inhibit CVB3 in Balb/c mice [126]. Two of the tested shRNAs exerted strong antiviral effects accompanied by attenuated pancreatic tissue damage. For both of the aforementioned studies, however, the siRNAs and shRNA expression plasmids, respectively, were applied by hydrodynamic transfection. This method involves high pressure injection of the nucleic acid into the tail vein, which does not lend itself to a therapeutic setting for humans. It was therefore necessary to develop alternative application routes for efficient RNAi-mediated inhibition of CVB3. Since delivery of chemically synthesized siRNAs across the endothelial barrier to cardiomyocytes is inefficient, even in the presence of transfection agents, transfer of shRNA expression cassettes by viral vectors appears to be the method of choice.

Kim *et al*. used a lentiviral vector to deliver the expression cassette for the above mentioned shRNA against the highly conserved CRE region [146]. Mice were injected intraperitoneally with the lentiviral vector and were subsequently challenged with CVB3. Treated animals had significant reductions in viral titers, viral myocarditis, and pro-inflammatory cytokines and, most importantly, the survival rate was improved from 20% to 50% at day ten after infection. Since retroviral vectors bear the risk of insertional mutagenesis and adenoviral vectors induce a strong immune response, vectors based on adeno-associated viruses (AAV) are considered promising vehicles for gene transfer. While the wild type virus is single-stranded, the more recently used viral vectors contain a self-complementary double stranded genome, which ensures rapid onset and a high level expression of the transgene [147]. Furthermore, tissue tropism of the viral vector can be directed by the use of pseudotyped AAV vectors, which consist of a genome based on the standard serotype 2 and capsid proteins from a different serotype. We could show that delivery of the shRNA double expression cassette by a pseudotyped, self-complementary AAV vector into primary rat cardiomyocytes inhibited CVB3 replication by about 3 orders of magnitude. Moreover, in mice with CVB3 myocarditis, the RNAi treatment significantly attenuated cardiac dysfunction [120].

Taken together, the *in vitro* studies demonstrate that RNAi is an efficient approach to inhibit CVB3 and subsequent *in vivo* studies confirmed viral vectors to be suitable vehicles for the delivery of shRNA expression cassettes to the heart. The technology thus has the potential to develop into a therapeutic option to treat humans with virus-induced myocarditis [148].

## 6. Soluble Receptors Analogues

A prerequisite of successful virus uptake is the specific binding of the virus to a cellular receptor. Soluble receptors analogues (SRA) bind to the virus before the virus interacts with their cellular receptors, thus preventing binding of the virus and subsequent uptake into the target cells. SRA can be found as naturally occurring cellular proteins generated by alternative splicing of the cellular receptor transcripts or are artificially designed as recombinant proteins by genetic engineering [149,150]. Many SRA lack the transmembrane domain. This domain is necessary for anchoring the natural receptor protein in the cell membrane and its absence enables SRA to pass through the cell membrane and accumulate in the extracellular space. In general, SRA that exclusively consist of virus binding sites are sufficient to neutralize the target virus. Their efficiency, however, is sometimes too poor to allow their use in therapeutic treatments [151,152]. Genetic engineering can increase SRA efficiency. The most common modification represents the fusion of the virus binding domain with the carboxy-terminus of the human IgG1 Fc region, resulting in generation of a dimeric antibody-like molecule [153]. Important effects result from this modification. The antibody Fc-region promotes the solubilization of the SRA, the half-life of the fusion protein increases up to 100-fold *in vivo* [154] and recognition of the IgG-Fc domain by Fc receptors expressed on the surface of macrophages enables clearing of the virus shortly after the virus is bound to the soluble receptor peptide by phagocytic cells [155]. Moreover, large amounts of soluble IgG-Fc can easily be purified using a protein A-coupled sepharose [150]. In this context it was shown that CVB3 neutralization was 125-fold more efficient with soluble DAF-IgG Fc fusion proteins (sDAF-Fc) than with the monomeric sDAF homologue comprising only of the cellular DAF ectodomain [156]. Alternatively the virus binding domain of a virus receptor can be fused to the C-terminal part of the C4b binding protein (C4bp) α chain leading to production of a disulfide-linked homo-octamer soluble protein with a spider-like structure [151]. Christiansen *et al*. reported that a fusion protein encompassing the CD46 ectodomain linked to C-terminal part of the C4bp α chain (sCD46-C4bpα) was able to bind to the measles virus hemagglutinin protein expressed on murine cells with a higher avidity than soluble monomeric CD46 (sCD46). Moreover, sCD46-C4bpα, but not the sCD46, fully protected CD46 transgenic mice against a lethal intracranial measles virus challenge [151]. 

**Figure 4:**
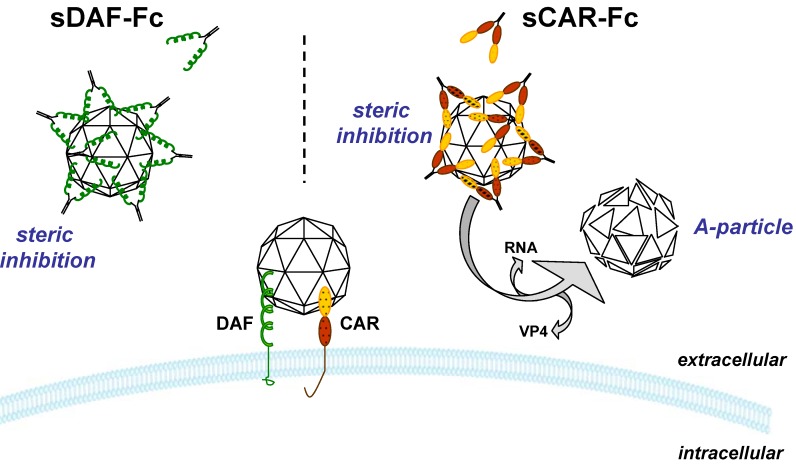
Mechanisms of inhibition of CVB3 by sCAR-Fc and sDAF-Fc. sCAR-Fc and sDAF-Fc bind to the viral capsid. This occupies the virus’s binding site for the receptors, preventing binding, and causes steric problems during the entry or uncoating steps for those which do manage to bind. Additionally, sCAR-Fc induces the formation of altered “A”-particles lacking VP4 and the viral RNA. A-particles are not infectious.

Competitive inhibition, as well as steric problems during the entry or uncoating steps, seem to be the main mechanisms responsible for blocking of viruses by SRA [157]. Another significant blocking mechanism was elucidated for SRA targeting viruses of the picornavirus family. In analogy to the physiological mechanism occurring during natural virus binding to cell receptor, it has been shown that the exposure of coxsackieviruses to virus specific SRA can induce the formation of altered (A)-particles [156,158] (Figure 4). A-particles have low buoyant density and lack the viral VP4 and the RNA-genome. These particles have lost their capacity to infect cells and their formation is an irreversible process [132,157]. 

The protective effect of SRA has been described for several viruses including HIV [159], adenovirus [160], measles virus [152] and human herpesvirus 6 (HHV6) [161]. Its effectiveness was also demonstrated for several members of the *picornoviridae* including entererovirus 71 [162], rhinovirus [157], and polioviruses [163]. To test their potential for inhibition of coxsackievirus myocarditis, SRA of CAR (sCAR-Fc) and DAF (sDAF-Fc) were developed and analyzed in different CVB3 myocarditis models [150,154,160,164]. Yanagawa *et al*. injected recombinant sDAF-Fc in mice 3 days before, concurrent with or 3 days after CVB3 infection. Histological examination demonstrated a significant reduction in lesion area, cell necrosis, calcification, and inflammation in hearts of animals treated before and concurrent with CVB3 but not in the post-infection group. However, all groups had a similar reduction of infectious CVB3 in the heart [150]. Very similar results were subsequently obtained with sCAR-Fc. The therapeutic effect induced by sCAR-Fc, however, seemed to be stronger than that observed for sDAF-Fc, especially in the pre- and concomitant treated groups [154]. Moreover, sCAR-Fc inhibited CVB3 induced pancreatitis *in vivo*, which was not found for sDAF-Fc [150,154]. Differences in efficiency can be explained by the fact that sCAR-Fc bind to CVB3 with 5,000- to 10,000-higher affinities than the equivalent form of DAF [156] but also the fact that sCAR-Fc induces A-particles, whereas sDAF-Fc forms reversible complexes with CVB3 [158], may contribute to this observation (Figure 4). 

Although these results convincingly demonstrate that sDAF-Fc and sCAR-Fc can efficiently inhibit CVB3-induced myocarditis, a comprehensive assessment regarding the impact on contractile heart parameters, the most important parameters from the clinical perspective, was not carried out in these studies. We therefore aimed at characterizing the protective effect of sCAR-Fc treatment in more detail. To this end, we cloned sCAR-Fc and inserted it into an adenoviral vector under control of a doxycycline (Dox) inducible promoter. Following systemic application, sCAR-Fc expression was induced by addition of Dox 2 days before, concomitant and 1 day after CVB3 infection. Induction of sCAR-Fc prior to infection completely blocked the virus and even concomitant and post-infection induction strongly reduced cardiac CVB3 infection, myocardial injury, and inflammation. Moreover, a significant improvement of systolic and diastolic cardiac parameters was detected in animals in which sCAR-Fc expression was induced prior to or concomitant with the CVB3 infection [164]. As Dox induction results in therapeutic serum levels of sCAR-Fc (ranging between 20–100 ng/mL [160]) 16 to 24 hours after induction at the earliest, the improvement in hemodynamic parameters in the concomitant group revealed that sCAR-Fc had a distinct therapeutic effect on heart function.

In a further approach aimed at generating sCAR-Fc/sDAF-Fc variants with increased efficiency, Lim *et al*. constructed sCAR-sDAF receptor chimeras containing the virus binding sites from hCAR and hDAF fused to the human IgG1 Fc region. One of this fusion proteins, hCAR:hDAF-Fc, showed stronger CVB3 neutralizing activity than sCAR-Fc *in vitro* and its employment *in vivo* resulted in significantly higher survival rates of CVB3 infected mice than observed for sCAR-Fc [160]. Finally, one study analyzed the potential of the naturally occurring sCAR variant CAR4/7, originally detected as a CAR splice variant in HeLa cells [149], for inhibition of CVB3 myocarditis. Although the viral load in hearts of CVB3 infected animals treated with CAR4/7 was significantly reduced, surprisingly the animals developed signs of autoimmune myocarditis characterized by aggravated myocardial inflammation, tissue damage and presence of CAR-specific antibodies which were autoreactive against myocardial tissues [165]. The mechanisms leading to these auto-immunologic side effects induced by CAR4/7 are not yet understood. There may be specific immunological reactions related to the specific sequence of CAR4/7, or perhaps it relates to the fact that recombinant CAR4/7 was produced in bacteria, which altered its glycosylation pattern and increased the risk of co-purifying pro-inflammatory substances. In this regard it should be noted that the other approaches employing sCAR or sDAF variants in CVB3 infected mice *in vivo* did not lead to these side effects, as mentioned above, demonstrating that it is not a general feature of sCAR, sDAF.

The therapeutic potential of the combination of sCAR-Fc or sDAF-Fc with other therapeutical approaches (pharmacologically active low molecular weight substances, RNAi, immunomodulators) in CVB3 myocarditis remains to be uncovered. As one initial example, we have recently demonstrated that the simultaneous application of sCAR-Fc and siRNAs against CVB3 exerts synergistic antiviral activity in the treatment of a persistently infected cardiac cell line *in vitro* [166]. Further studies are currently underway to verify these results in CVB3 myocarditis models *in vivo*.

Summarizing these data, it can be concluded that sCAR-Fc and sDAF-Fc represent novel promising biologics that may potentially be used for treatment of coxsackievirus infections in humans. However, several questions are still unanswered and must be addressed in further research. Can coxsackieviruses develop resistance against sCAR and sDAF? What is the safety profile after long term application? Can sCAR or sDAF inhibit the development of chronic forms of CVB3 myocarditis?

## 7. Cell Therapy

Due to the great progress made in the understanding of stem cell therapy in recent years, application of stem cells is considered as one of the most promising strategies for therapeutic interventions in the future. Regenerative approaches aim at the restoration of the physiological cellular composition of diseased organs. Recent research indicates that the mammalian heart can be repopulated by cells from extra-cardiac sources. Transplantation of exogenous cells is therefore considered to be the likely next generation of cardiac cell therapies [167].

Mesenchymal stem cells (MSC) have the particular advantage of being non-immunogenic and thus allow the use of allogenic MSCs for clinical applications. In a recent preclinical study, the potential of MSCs to improve myocarditis induced by CVB3 was demonstrated for the first time [168]. As a prerequisite for the clinical application of this strategy, MSCs were shown not to be infected by CVB3, most likely due to a low expression level of CAR. In co-culture experiments with the cardiomyocyte cell line HL-1, MSCs reduced CVB3-induced apoptosis and oxidative stress. Furthermore, MSC diminished viral progeny release by approximately 5-fold. Importantly, intravenous injection of MSCs decreased cardiac apoptosis and improved left ventricular function in an experimental model of murine acute CVB3-induced myocarditis. A detailed mechanistic analysis revealed that the protective effect of the MSCs is mediated in an NO-dependent manner and requires priming via IFN-γ. Further research, however, will be required to investigate possible side effects and the potential of MSC transplantation for the treatment of CVB3 induced myocarditis.

## 8. Concluding Remarks

Coxsackieviruses are one of the most important infectious agents associated with acute and chronic myocarditis in humans. Based on the elucidation of the coxsackieviral replication cycle a wide panel of pharmacologically active low molecular weight substances with strong antiviral activity have been developed but to date none are in routine clinical use. More recently biologics such as SRA, siRNAs or MSC have been found to be suitable to inhibit viral replication and inhibit coxsackievirus myocarditis (Table 1), but further investigation is necessary to elucidate their full therapeutic potentials and safety profiles. 

**Table 1:** Summary of antiviral therapeutics used for treatment of coxsackievirus infections.

*in vitro*	*in vivo*	*clinical treatment*
**compounds interacting with**	**compounds interacting with**	**compounds interacting**
**viral capsi**	**viral capsid**	**with viral capsid**
*different WIN compounds*	*WIN 54954*	*WIN 63843 (Pleconaril)*
	*WIN 63843 (Pleconaril)*	
**compounds interacting with**		
**viral protein 2C**		
*Guanidine hypochlorid,*		
*HBB, MRL-1237,*		
*TBZE-029*		
**compounds interacting with**	**compounds interacting with**	
**viral proteases 2A and 3C**	**viral proteases 2A and 3C**	
***NO-donor*** *: GTN, ISDN*	***NO-donor*** *: GTN,*	
	*NO-metoprolol*	
**nucleoside analogues**	**nucleoside analogues**	
*5-nitrocytidine*	*Ribavirin*	
**UPS-Inhibitors**	**UPS-Inhibitors**	
*PDTC, Curcumin,*	*MLN 353*	
*MG132, Lactacystin*		
**Interferons**	**Interferons**	**Interferons**
*IFN-β, IFN-γ, IFN-α*	*IFN-β, IFN-α*	*IFN-β (Betaferon®*)
**Antisense oligonucleotides**	**Antisense oligonucleotides**	
**siRNA and shRNA**	**siRNA and shRNA**	
**soluble receptor analogues**	**soluble receptor analogues**	
*sDAF-Fc*	*sDAF-Fc*	
*sCAR-Fc*	*sCAR-Fc*	
*sCAR-sDAF-Fc*	*sCAR-sDAF-Fc*	
**cell therapy**	**cell therapy**	
*MSC*	*MSC*	

Based on our understanding of the pathogenesis of coxsackievirus-induced myocarditis, considering that both viral and immune and autoimmune mechanisms are involved in progression of the disease, antiviral therapy and its combination with immunomodulatory therapies may hold the greatest potential for improving the therapeutic outcome of coxsackievirus infection. 
